# A Multiple Antigenic Peptide Mimicking Peptidoglycan Induced T Cell Responses to Protect Mice from Systemic Infection with *Staphylococcus aureus*

**DOI:** 10.1371/journal.pone.0136888

**Published:** 2015-08-28

**Authors:** Xiang-Yu Wang, Zhao-Xia Huang, Yi-Guo Chen, Xiao Lu, Ping Zhu, Kun Wen, Ning Fu, Bei-Yi Liu

**Affiliations:** 1 Department of Immunology, School of Basic Medical Sciences, Southern Medical University, Guangzhou, Guangdong, People’s Republic of China; 2 Laboratory of Emerging Infectious Diseases and Division of Laboratory Medicine, Zhujiang Hospital, Southern Medical University, Guangzhou, Guangdong, People’s Republic of China; Wayne State University, UNITED STATES

## Abstract

Due to the enormous capacity of *Staphylococcus aureus* to acquire antibiotic resistance, it becomes imperative to develop vaccines for decreasing the risk of its life-threatening infections. Peptidoglycan (PGN) is a conserved and major component of *S*. *aureus* cell wall. However, it has not been used as a vaccine candidate since it is a thymus-independent antigen. In this study, we synthesized a multiple antigenic peptide, named MAP27, which comprised four copies of a peptide that mimics the epitope of PGN. After immunization with MAP27 five times and boosting with heat-inactivated bacterium one time, anti-MAP27 serum bound directly to *S*. *aureus* or PGN. Immunization with MAP27 decreased the bacterial burden in organs of BALB/c mice and significantly prolonged their survival time after *S*. *aureus* lethal-challenge. The percentage of IFN-γ^+^CD3^+^ T cells and IL-17^+^CD4^+^ T cells in spleen, as well as the levels of IFN-γ, IL-17A/F and CCL3 in spleen and lung, significantly increased in the MAP27-immunized mice after infection. Moreover, *in vitro* incubation of heat-inactivated *S*. *aureus* with splenocytes isolated from MAP27-immunized mice stimulated the production of IFN-γ and IL-17A/F. Our findings demonstrated that MAP27, as a thymus-dependent antigen, is efficient at eliciting T cell-mediated responses to protect mice from *S*. *aureus* infection. This study sheds light on a possible strategy to design vaccines against *S*. *aureus*.

## Introduction

*Staphylococcus aureus* (*S*. *aureus*), one of the most common Gram-positive bacterium, causes a series of life-threatening diseases including sepsis, bacteremia and necrotizing pneumonia etc [1, 2]. In recent years, the treatment has become more and more difficult for infections caused by methicillin-resistant *S*. *aureus* (MRSA), strains that are resistant to almost all the commonly used antibiotics [3]. This fact urges pharmaceutical companies to develop new and effective prophylactic vaccines and immunotherapies in the battle against the bacterium. However, attempts at making *S*. *aureus* vaccines have so far been unsuccessful [4, 5]. This failure has been attributed partly to the feature of the bacterium, which exhibits diverse arrays of virulence factors among individual clinical isolates, and can change its surface antigen repertoire potentially in different infection states [5]. Thus, it would be wise to select a conserved antigen that is expressed on bacterium persistently and steadily as a vaccine candidate. The second obstacle for development of *S*. *aureus* vaccine is that the relationship between the bacterium and the immune system remains ambiguous. *S*. *aureus* can evade the host immune system by inhibiting phagocytic uptake and its killing activity [6, 7]. Moreover, the effect of antibody-mediated opsonization is controversial [4, 5, 8]. Recently, more and more reports indicate that T cell-mediated immune responses, especially stimulating and secreting cytokines such as IL-17 or IFN-γ, might play an important role in prevention of *S*. *aureus* infection [9–17].

Peptidoglycan (PGN), accounting for about 50 percent weight of the bacterium wall, is made of polymeric meshwork of glycan strands crosslinked by short peptides [18]. In bacterium, PGN maintains the cell shape and osmotic pressure of cytoplasm. Therefore, it is a crucial and conservative component of *S*. *aureus*. However, as a thymus-independent antigen (TI antigen) with weak immunogenicity, PGN is not considered as a rational vaccine candidate. Mimetic peptides, acting as a thymus-dependent antigen (TD antigen) with relatively stronger immunogenicity, have been used as a promising surrogate for carbohydrate to generate vaccines. Immunization with a peptide, mimicking a carbohydrate antigen of tumor cells or polysaccharide in bacterium, could augment tumor-specific cellular responses or induce protective antibody responses to bacterium [19, 20]. Recently, we successfully obtained a series of PGN-mimicking peptides from phage peptide library by using an anti-PGN mAb as the target [21]. In this study, we synthesized a multiple antigenic peptide (MAP), named MAP27, which carried four copies of an identical peptide derived from the screening of the phage peptide library. Immunization with MAP27 decreased the bacterial burden in organs of the mice and significantly prolonged their survival time after *S*. *aureus* lethal-challenge. This protection is mainly correlated with the induction of IFN-γ- and IL-17A- producing T cells by MAP27 immunization.

## Materials and Methods

### Mice and Ethics Statement

BALB/c mice (4–6 weeks of age, female) were purchased from the Experimental Animal Center, Southern Medical University, Guangzhou, China. All of the animal experiments were approved by the Animal Ethical and Experimental Committee of the Southern Medical University (permit number: 2014–028). All mice were maintained under specific-pathogen-free conditions in a clean room at the Institute for Animal Experimentation in Southern Medical University. They were kept on a 12:12 h light-dark cycle with food and water provided *ad libitum*. Animal experiments were carried out in strict accordance with national guidelines for animal welfare. To alleviate pains, all mice were anesthetized with sodium pentobarbital (50 mg/kg) via abdominal cavity before *S*. *aureus* infection. According with the principle of animal ethics, the humane euthanasia was performed. During the bacterial lethal challenge, mice were observed every 2 hours for the first 48 hours, and the survival was monitored for at least 7 days. Mice that became moribund (such as hunched back, ruffled fur and lethargy), the survivors at the endpoint of observation during *S*. *aureus* lethal challenge, and the mice infected with *S*. *aureus* for three days were sacrificed by CO_2_ asphyxiation.

### Peptide Synthesis

All peptides, including three linear peptides (SP27, SP27’ and L2), a multiple antigenic peptide (named MAP27), and the attaching backbone of MAP (MAPctrl) were synthesized by Hybio Pharmaceutical (Shenzhen, China). Sequence accuracy of these peptides was confirmed by mass spectrometry and high performance liquid chromatography. Peptide purity was above 95% and the residual endotoxin was measured below 0.025 EU/ml. All of the peptides, provided as lyophilized powders, were dissolved in endotoxin-free water (Sigma) at 20mg/ml and stored at -70°C.

### The antigenicity of linear peptides and MAPs

For examination of the antigenicity of linear peptides, enzyme linked immunosorbent assay (ELISA) was performed as described by Elshabrawy *et al*. [22, 23]. Briefly, 96-well plates were coated with 5 μg/ml monoclonal antibody (mAb) against PGN (AbD Serotec, MCA5792, clone No.11-232.3, isotype: mouse IgG3) or mAb against Lipoteichoic acid (LTA, Thermo scientific, MA1-7401, clone No. G35C, isotype: mouse IgG1) at 4°C for overnight. Biotin-labeled SP27, SP27’ or L2 as a control peptide (sequence: Biotin-HSGHWDFRQWWQPSGG) was added to the wells, followed by detection with HRP-labeled streptavidin (Jackson ImmunoResearch, 1/10000 dilution). For the examination of MAP27, 5 μg/ml anti-PGN mAb or anti-*S*. *aureus* polyclonal antibodies (stored at our laboratory, 1/800 dilution) were added to the wells coated with 80 μg/ml MAP27 or MAPctrl, and incubated at 37°C for 40 min. HRP-labeled goat anti-mouse and goat anti-rabbit IgG (Jackson ImmunoResearch, 1/10000 dilution) were then added (37°C for 40 min), respectively. Wells were washed three times with 0.1% PBS/Tween 20, followed by addition of the substrate solution containing TMB and H_2_O_2_ for color reaction.

### Preparation of bacteria

*S*. *aureus* (ATCC 25923) were purchased from Wenzhou Kont Biology and Technology. The bacteria were grown in tryptic soy broth at 37°C with 250 rpm shaking for overnight. After centrifugation at 6000 rpm for 10 min, the pellet was resuspended and subsequently washed with sterile phosphate-buffer saline (PBS) for two times. The pellet was then diluted with PBS to an appropriate cell concentration as determined by spectrophotometry at 600 nm.

### Immunization and ELISA for specific antibodies

The protocol of vaccination was based on our previous study with slight modifications [21]. Briefly, female BALB/c mice were divided randomly into three groups. The first two groups were injected subcutaneously with 100 μg MAP27 and 100 μg MAPctrl for five times at 2-week intervals, respectively. The first immunization was performed in Freund’s complete adjuvant (Sigma) and the subsequent booster immunizations were administered in Freund’s incomplete adjuvant (Sigma). The third group of mice, without any peptide immunization, was breed at the same time and used as the blank control. After 14 days of the fifth MAP immunization, all of the three mice groups (MAP27-immunized, MAPctrl-immunized, and blank control mice) were finally immunized with heat-inactivated *S*. *aureus* (2×10^7^ CFU/mouse in sterile PBS without any adjuvant) via intraperitoneal injection. Serum samples were collected through retro-orbital bleeding 7 days after each immunization and frozen at -70°C for antibody evaluations.

Antibody titers and specificity were measured by ELISA as described previously [21]. Briefly, MAP27 (80 μg/ml), sonicated fragments of *S*. *aureus* (OD_600nm_ = 0.5, 50 μl/well) and PGN (10 μg/ml) were coated in 96-well plates at 4°C for overnight, respectively. MAP27- and PGN-coated plates were blocked with 0.25% casein, whereas *S*. *aureus* fragments-coated plate were blocked with blocking buffer containing 1% guinea pig serum to avoid binding of staphylococcus protein A to IgG. The serial diluted antiserum was subsequently added as the primary antibodies, followed by incubation with HRP-conjugated goat anti-mouse IgG as the secondary antibodies. The titer of antibody was defined as the highest dilution that gives more than twice the absorbance value of the blank control.

### Mice challenged with lethal live *S*. *aureus*

Five days after the final boost, all of the mice were infected with *S*. *aureus* (ATCC 25923) at 5×10^8^ CFU/mouse via tail vein injection. The mortality was monitored for 7 days (168 hours) post challenge.

### Measurement of bacterial burden in organs

To determine the bacterial loads in organs, all of the three groups of mice were infected with *S*. *aureus* at 2×10^7^ CFU/mouse via tail vein. Three days after infection, mice were sacrificed by CO_2_ asphyxiation. The unilateral kidney and lung were aseptically removed and homogenized with 2 ml sterile PBS. The homogenates were then plated on agar media in a 10-fold serial dilution and incubated at 37°C for 18–24 h. The colony forming units (CFU) was determined.

### Levels of cytokines in organs post *S*. *aureus* systemic infection

Mice were euthanized by CO_2_ asphyxiation three days post infection. To measure the local inflammatory response, we centrifuged the homogenized lysates from spleen and lung. Supernatant were collected, added with protease inhibitors (Cell Signaling Technology) and analyzed by ELISA (Biolegend) according to the manufacturer’s instructions.

### Intracellular cytokine analysis

Cytokines produced by CD3^+^ T cells were detected by flow cytometry based on a modified method as described by Chen *et al*. [24] and Bhattacharya *et al*. [25]. Briefly, the splenocytes were harvested three days post infection with *S*. *aureus*. 1–2×10^6^ lymphocytes were incubated with cell stimulation cocktail (containing phorbol 12-myristate 13-acetate, PMA and ionomycin, eBioscience) and brefeldin A (BFA, eBioscience) for 6 h. Cells were then washed, stained with fluorescein-labeled antibodies (FITC labeled anti-CD3 mAb, Cat No. 11–0031, clone:145-2C11; APC labeled anti-CD4 mAb, Cat No.17-0042, clone:RM4-5; eBioscience). Following the fixation and permeabilization, PE-conjugated anti-mouse interferon gamma (IFN-γ) mAb (Cat No.12-7311, clone: XMG1.2, eBioscience) or interleukin 17A (IL-17A) mAb (Cat No. 12–7177, clone: eBio17B7, eBioscience) was used to measure the intracellular cytokine production in CD4^+^ T cells or CD4^-^ T cells, respectively. Data were acquired with a FACS Calibur (BD, Biosciences, San Jose, CA, USA) and analyzed using FCS Express software (De Novo Software, Los Angeles, CA USA). Isotype controls were included in each staining.

### *In vitro* stimulation of splenocytes

Five days after the last heat-inactivated *S*. *aureus* boost, splenocytes from MAP27-immunized, MAPctrl-immunized and blank mice were obtained by squeezing followed by filtering through a stainless mesh (size, 70 μm) in RPMI1640 medium. Erythrocytes were lysed with RBC lysis buffer (eBioscience). After being washed two times with RPMI1640, cells were resuspended with complete medium (RPMI1640 containing 10% fatal calf serum (Gibico), 100 U/ml of penicillin G and 100 μg/ml of streptomycin) in 96-well U-bottom culture plates. The splenocytes (2 ×10^5^ cells/well) were incubated at 37°C with 100 μg/ml MAP27 for 24 h or 2×10^4^ CFU/well heat-inactivated *S*. *aureus* for 72 h. The supernatants were collected after the incubation and stored at -70°C. The amount of interleukin 2 (IL-2), INF-γ, IL-17A/F and interleukin 4 (IL-4) was determined by ELISA (Biolegend) according to the manufacturer’s instructions. All samples were analyzed in triplicate.

### ELISPOT assays

The IFN-γ and IL-17A spot ELISA assays were performed using a kit according to the manufacture’s instruction (eBioscience). Briefly, ELISPOT plates (Millipore) were coated with capture antibodies, and incubated at 4°C overnight. The plates were blocked with complete medium at room temperature for 1 h. Splenocytes were harvested and plated at 4×10^5^/well, stimulated with 10 μg/ml MAP27 plus 1μg/ml anti-mouse CD28 antibody or 2×10^5^ CFU/well heat-killed *S*. *aureus* at 37°C for 24 h. Cells and medium were decant from plates, and the plates were washed for three times with washing buffer. Biotinylated detection antibodies and HRP-conjugated avidin were added subsequently. The plates were extensively washed, and developed with substrate solution containing AEC (Sigma) and H_2_O_2_. The reaction was terminated with H_2_O. After drying, the plates were read using CTL analyzer (CTL S5 micro, Cellular Technology Ltd.).

### Statistical methods

Results were expressed as means ± SEM. Statistical analyses of data to compare the different groups were performed using SPSS version 16.0 software. Data for specificity of antibody, cytokine production and ELISPOT were analyzed using either one-way ANOVA or Student’s t-test. Data for flow cytometry and bacterial load were analyzed by Mann-Whitney U of nonparametric Test. For comparison of survival in murine lethal challenge model, individual experiments were analyzed using log-rank test statistic (Mantel-Cox test) from the Prism software (Prism for Windows, version 5.01, GraphPad Software). Probability (*P*) values <0.05 were considered significant. 4 to 9 mice were used in each experiment and each experiment was repeated two or three times with consistent results.

## Results

### Both linear peptides and tetra-branched, multiple antigenic peptide MAP27 bind to antibody specifically

**Fig 1:**
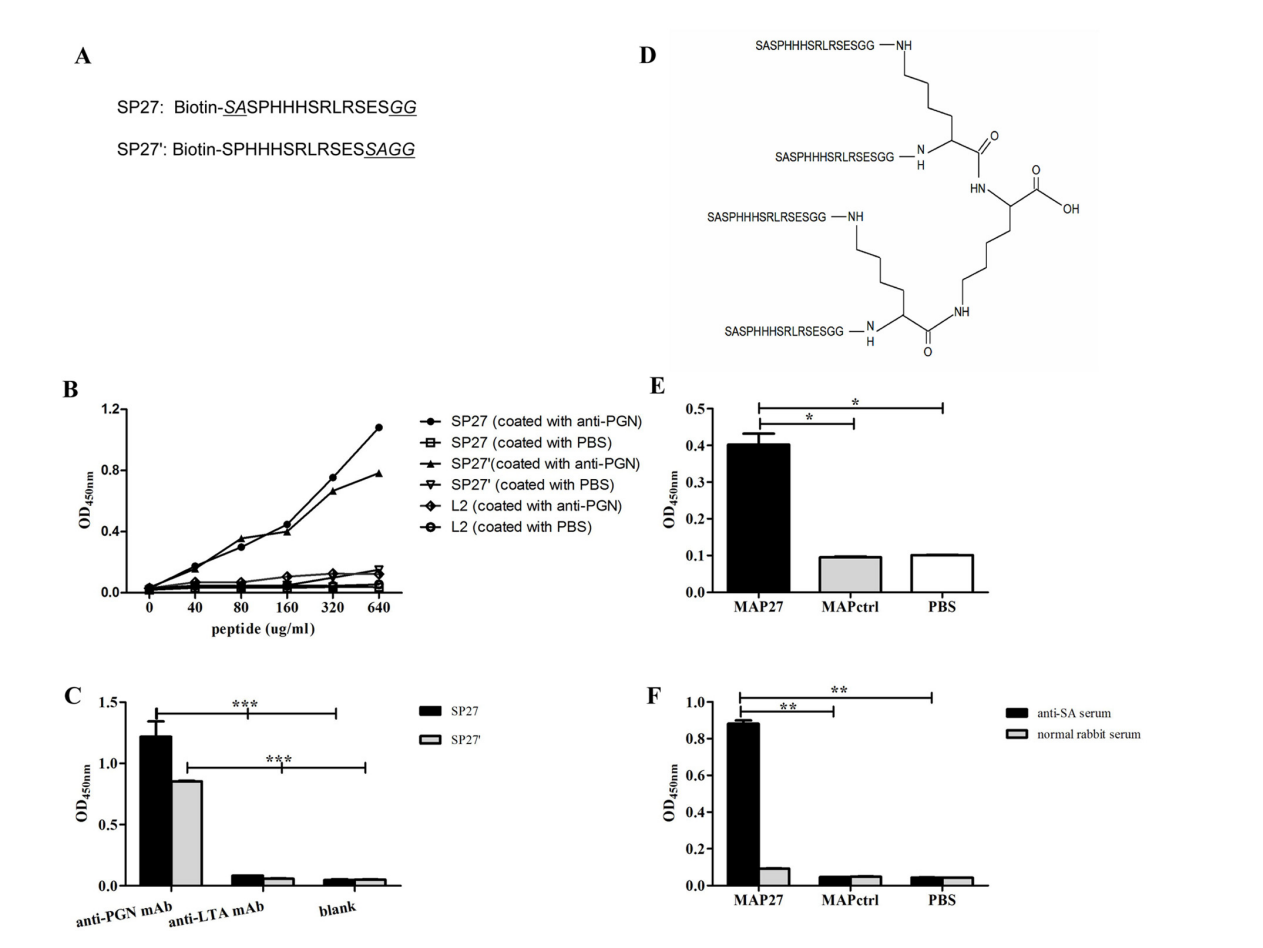
Both SP27 and MAP27 bind to anti-PGN mAb specifically. (A) The sequences of SP27 (Biotin-*SA*SPHHHSRLRSES*GG*) and SP27’ (Biotin-SPHHHSRLRSES*SAGG*). Underlined letters represent nonspecific flanking amino acids. (B) Both SP27 and SP27’ bind to anti-PGN mAb in a dose-dependent manner. (C) Both SP27 and SP27’ (100 μg/ml) specifically bind to anti-PGN mAb, but not to anti-LTA mAb. For ELISA assays in panel B and C, anti-PGN mAb or anti-LTA mAb was used to coat the wells at a concentration of 5 μg/ml, SP27, SP27’ or non-specific peptide L2 (in panel B, sequence: Biotin-HSGHWDFRQWWQPSGG) was then added at indicated concentrations and incubated at 37°C for 40 min, followed by detection with HRP-labeled streptavidin. (D) The structure diagram of MAP27. MAP27 was synthesized in a tetra-branched form that contains four copies of a sequence (SASPHHHSRLRSESGG) that mimics PGN epitope. (E) MAP27 binds to anti-PGN mAb specifically. (F) MAP27 binds to anti-*S*. *aureus* polyclonal antibodies specifically. For ELISA assays in panel E and F, MAP27 or MAPctrl was used to coat the wells of a microplate. Anti-PGN mAb or anti-*S*. *aureus* polyclonal antibodies was added, followed by detection with HRP-labeled antibodies. The absorbance was measured at OD_450nm_. The results are shown as means ±SEM. * P<0.05, ** P<0.01, *** P<0.001.

Both of the two linear peptides, SP27 (Biotin-*SA*SPHHHSRLRSES*GG*) and SP27’ (Biotin-SPHHHSRLRSES*SAGG*), contain the same core sequence SPHHHSRLRSES, flanked by 2–4 amino acids at N- and C- terminus (Fig 1A). The core sequence is derived from one of the positive phage clones we previously identified by using an anti-PGN mAb as the target [21]. As shown by ELISA, both SP27 and SP27’ can bind to anti-PGN in a dose-dependent manner. In contrast, a non-specific peptide L2 (Biotin-HSGHWDFRQWWQPSGG) did not bind to the anti-PGN mAb (Fig 1B). Moreover, neither SP27 nor SP27’ could bind to an unrelated anti- Lipoteichoic acid (LTA) mAb (Fig 1C). These results indicate that the core sequence (SPHHHSRLRSES), but not the flanking amino acids (SA, GG in SP27, or SAGG in SP27’) might mimic the epitope of PGN.

Compared with a linear peptide, a multiple antigenic peptide (MAP) has more advantages: it is more stable upon enzymatic degradation; it has bigger size that is sufficient for immunization without crosslinking with any carrier protein; moreover, MAP enhances molecular recognition by immune cells and induces stronger immune responses [26, 27]. Therefore, MAP has been used as an immunogen for developing experimental vaccines against various pathogens [28–32]. Based on this fact, we synthesized a MAP, named MAP27 using standard Fmoc chemistry. MAP27 contains four copies of SP27, with C-terminus of each peptide linked with the non-immunogenic, lysine-based dendritic scaffold (Fig 1D). The predicted molecular weight of MAP27 is 7137.7, big enough for immunization according to our previous work [21]. Compared with the attaching backbone (MAPctrl), MAP27 specifically bound to anti-PGN mAb (Fig 1E) and anti-*S*. *aureus* polyclonal antibodies (Fig 1F). These results indicate that a similar antibody-binding epitope as PGN was remained in MAP27.

### Last boost with heat-inactivated bacterium produced low titer antibody against *S*. *aureus* in MAP27- immunized mice

**Fig 2:**
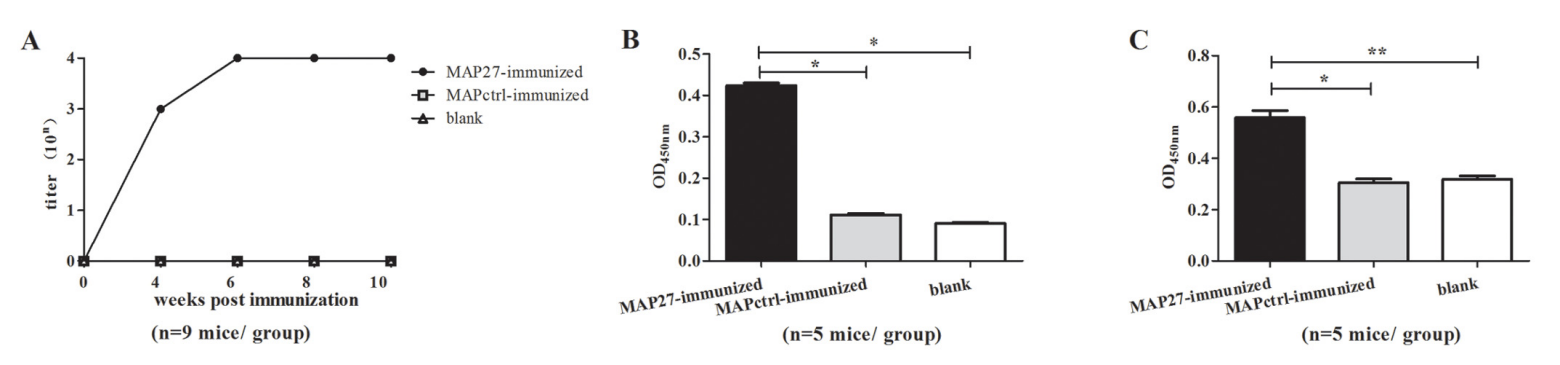
BALB/c mice immunized with MAP27 produced anti-MAP27, anti-PGN and anti-*S*.*aureus* antibodies. (A) Titers of anti-MAP27 serum during the period of immunization. 96-well plates were coated with MAP27. Serum samples from mice immunized with MAP27, MAPctrl or blank were pooled and added in a 10-fold serial dilution, followed by incubation with HRP-conjunctive goat anti-mouse IgG. (B) Sera from MAP27-immunized mice bind to PGN after the fifth MAP immunization. (C) Sera from MAP27-immunized mice bind to *S*. *aureus* after boosting with heat-killed *S*.*aureus*. For ELISA assays in panel B and C, 96-well plates were coated with PGN or sonicated *S*. *aureus* fragments. Serum samples were added in a 1/200 dilution as primary antibodies, followed by incubation with HRP-conjunctive goat anti-mouse IgG. The absorbance was measured at OD_450nm_. The results are presented as means ±SEM. * P<0.05, ** P<0.01, n = 5–9 mice/group.

We then evaluated the immunogenicity of MAP27. Serum samples from mice immunized with MAP27 or MAPctrl were used for binding assays by indirect ELISA. The titer of antibodies against MAP27 in MAP27-immunized mice reached to 10^4^ after the third time of vaccination, whereas no detectable antibodies were produced in MAPctrl-immunized mice (Fig 2A). We also found that serum from MAP27-immunized mice weakly reacted with PGN after the fifth MAP immunization (Fig 2B, at the 1/200 dilution). In contrast, antibodies to *S*. *aureus* were not detected in the same samples (data not shown). As shown in previous studies, boosting with natural antigens after immunization with peptide could significantly enhance the immune response [33, 34]. Therefore, we boosted all of the three groups of mice with heat-inactivated *S*. *aureus* for only one time. Anti-*S*. *aureus* IgG from MAP27-immunized mice could be detected with a dilution of 1/200. Conversely, the serum samples from either MAPctrl-immunized mice (P = 0.016) or blank control mice (P = 0.002) produced no detectable antibodies (Fig 2C). These results indicate that *S*. *aureus* boosting specifically augmented the immune responses triggered by MAP27 immunization.

### Vaccination with MAP27 protected the mice against *S*. *aureus* lethal-challenge and significantly reduced the bacterial burden in organs

**Fig 3:**
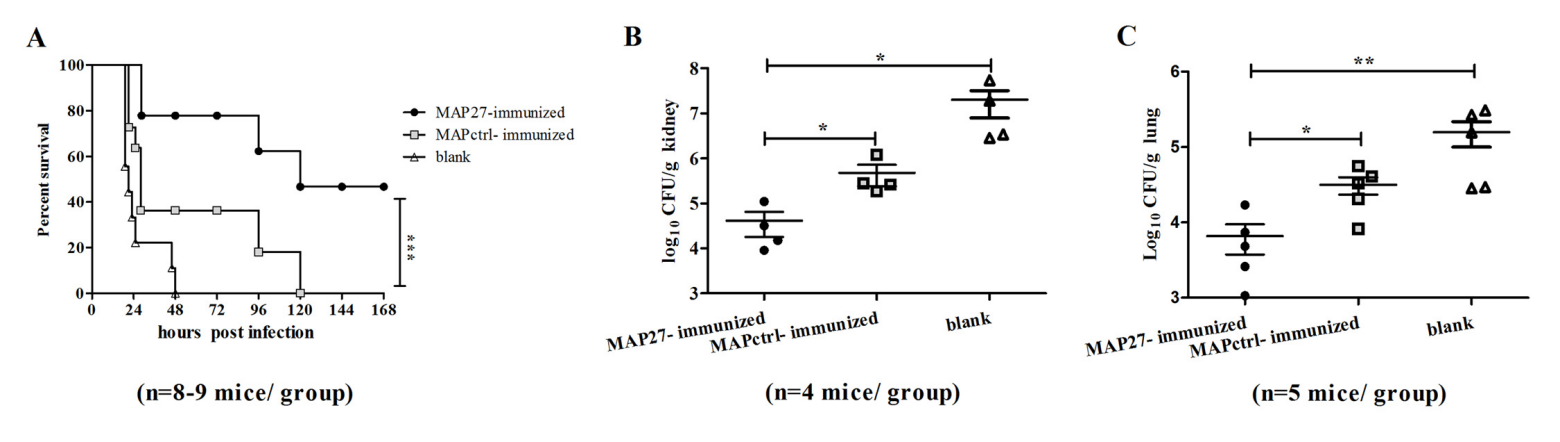
Immunization with MAP27 protects mice against *S*. *aureus* infection. (A) Survival analysis of mice with or without immunization after *S*. *aureus* infection. Five days after boosting with heat-inactivated *S*. *aureus*, all of BALB/c mice (including MAP27-immunized, MAPctrl-immunized and blank control, n = 8–9 mice/group) were challenged with *S*. *aureus* (ATCC 25923, 5×10^8^ CFU/mouse) by intravenous injection. Survival rate were monitored for seven days after infection. (B) Bacterial numbers in kidney from three groups of mice (n = 4 mice per group) were measured after three days infection of *S*. *aureus* (2×10^7^ CFU/mouse). (C) Bacterial numbers in lung from different groups of mice (n = 5 mice per group) were measured after three days infection. * P<0.05, ** P<0.01, *** P<0.001.

We then tested whether MAP27 immunization protects the mice against *S*. *aureus* infection. All of the mice were challenged with live *S*. *aureus* at a lethal dose on the fifth day post the last boost of heat-inactivated bacterium. The survival was then monitored. After seven days of lethal challenge, 50% of the MAP27-immunized mice were still alive, whereas, all of the control mice and MAPctrl-immunized mice died on day two (P = 0.000) and day five (P = 0.000), respectively (Fig 3A). This result indicates that MAP27 vaccination protects the mice against *S*. *aureus* lethal challenge.

To further determine the effect of MAP27 immunization on bacterial growth in animal organs, all of the mice were sacrificed three days after *S*. *aureus* infection. We found that the number of bacterium in the kidney of MAP27-immunized mice is significantly fewer than that of MAPctrl-immunized mice (P = 0.029) or blank control mice (P = 0.029) (Fig 3B). Similar results were also observed in lung (Fig 3C, MAP27 vs MAPctrl, P = 0.016; MAP27 vs blank control, P = 0.008). Taken together, these experiments indicate that vaccination with MAP27 reduced bacteria burden in animal organs.

### IFN-γ, IL-17A/F and CCL3 were increased in organs of MAP27-immunized mice after *S*. *aureus* systemic infection

**Fig 4:**
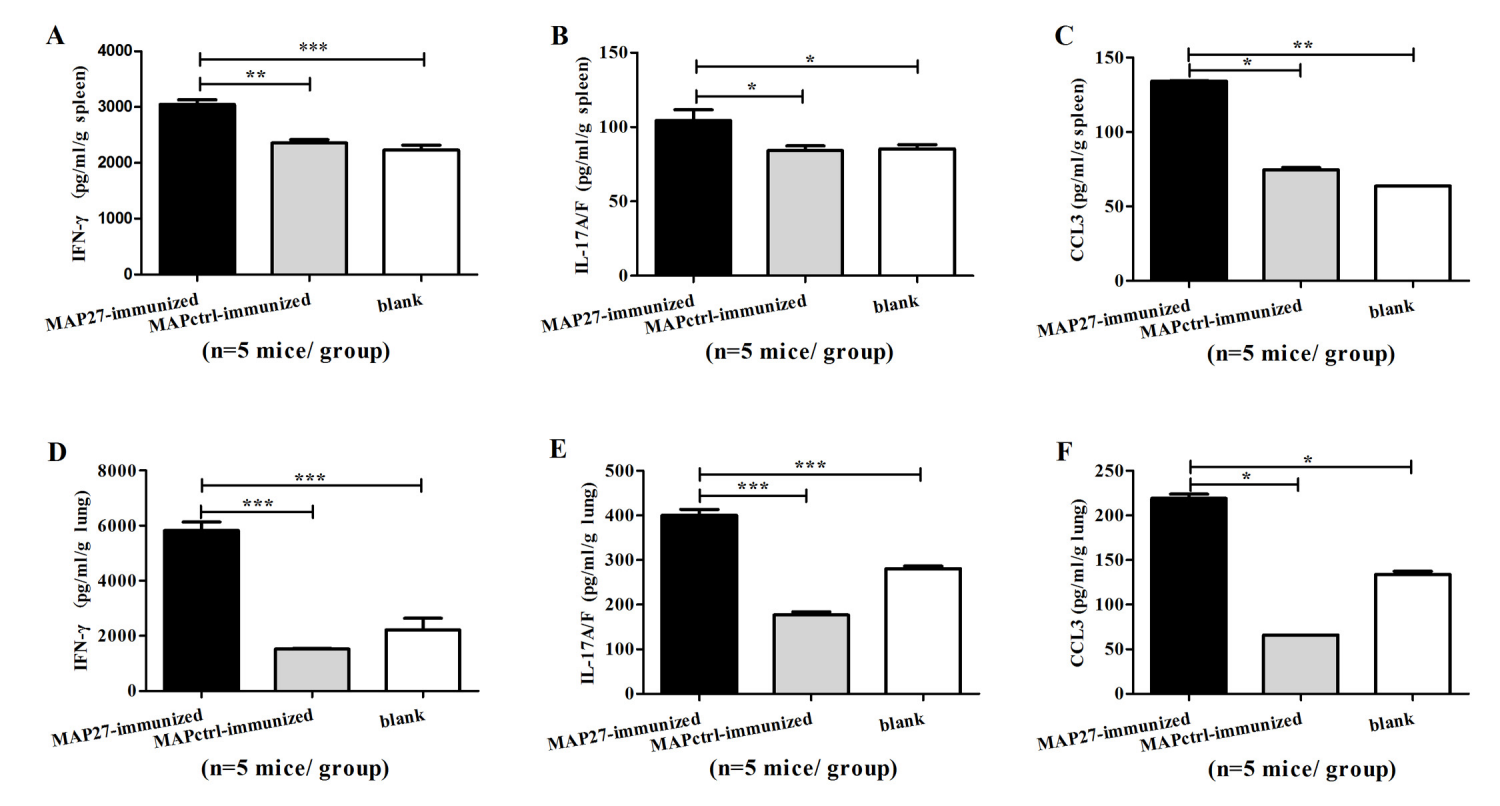
Immunization with MAP27 promoted IFN-γ, IL-17A/F and CCL3 production in organs post infection with *S*. *aureus*. Five days after boosting with heat-inactivated *S*. *aureus*, all of the mice (n = 5 mice per group) were infected with *S*. *aureus* (2×10^7^ CFU/mouse) for three days. Spleen and lung were aseptically taken and homogenized with sterile PBS. The supernatants were pooled and analyzed by ELISA. The concentrations of IFN-γ (panel A and D), IL-17A/F (panel B and E), and chemokine ligand 3 (CCL3, panel C and F) were measured in spleen and lung, respectively. * P<0.05, ** P<0.01, *** P<0.001.

As we described above, although MAP27 immunization only induced the production of low titer antibodies against *S*. *aureus* or PGN, it protected the mice against *S*. *aureus* systemic infection. This fact led us to speculate that the protection effect is possibly through the *in vivo* T cell-mediated response. To test this hypothesis, we examined T cell-derived cytokines in spleen and lung from the mice three days post infection. As expected, the levels of IFN-γ and IL-17A/F, cytokines mainly secreted by T cells, were remarkably higher in MAP27-immunized mice than in MAPctrl-immunized mice and blank control mice (Fig 4). CCL3, a chemokine that activates macrophages and neutrophils [35, 36], as well as enhances IFN-γ production [37], was also increased in MAP27-immunized mice (Fig 4). These results indicate that T cell responses might mediate the protection of MAP27 immunization against the *S*. *aureus* systemic infection.

### The IFN-γ^+^CD3^+^ and IL-17A^+^CD4^+^ T cells were increased in spleen of MAP27-immunized mice post *S*. *aureus* systemic infection

**Fig 5:**
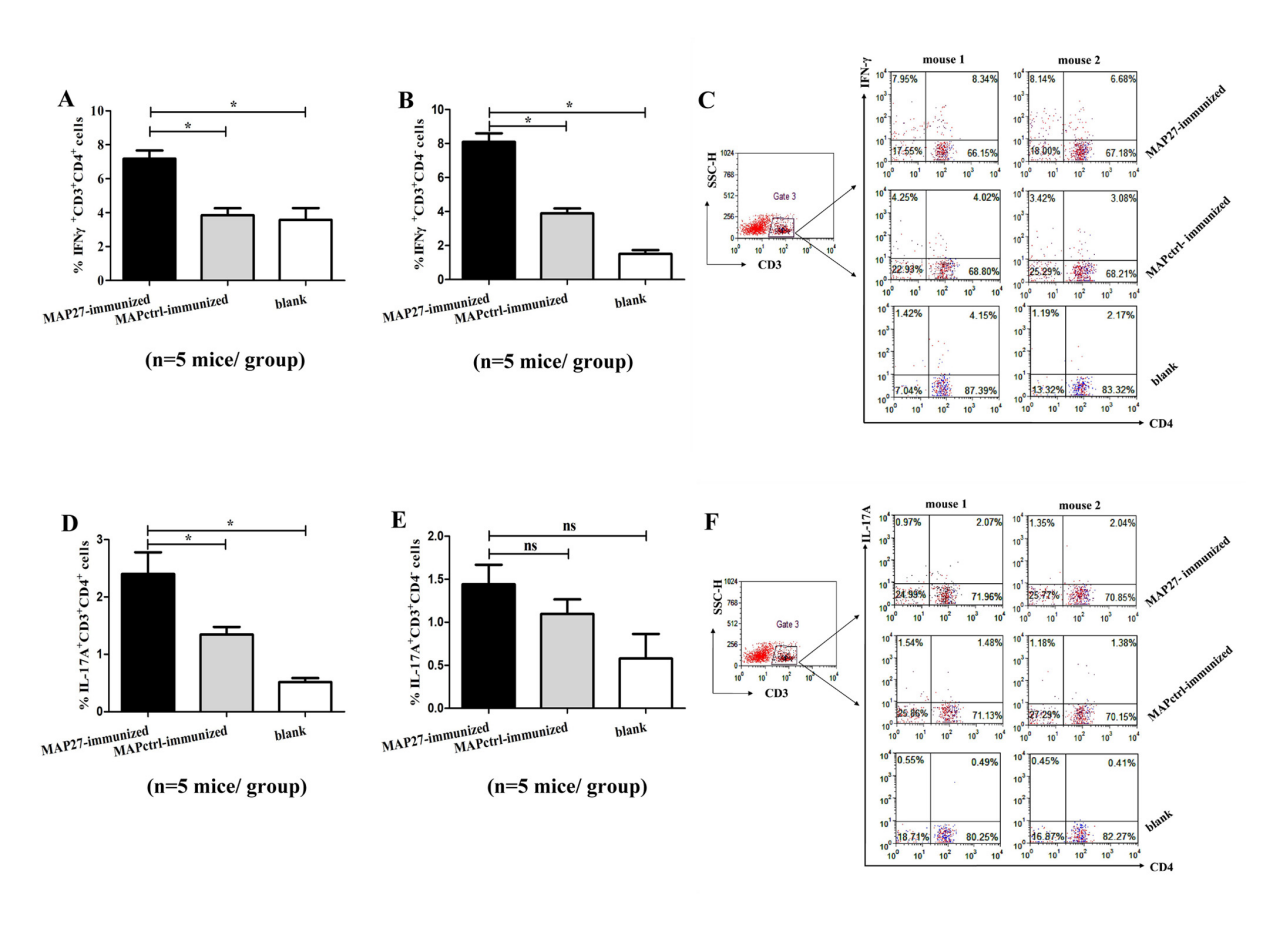
Vaccination with MAP27 primed IFN-γ^+^CD3^+^ T cells and IL-17^+^CD4^+^ T cells after *S*. *aureus* infection. Five days after the last boost with heat-inactivated *S*. *aureus*, all of the mice were infected with *S*. *aureus* (2×10^7^ CFU/mouse). Spleen cells (n = 5 mice per group) were harvested three days post infection and incubated with cocktail (including PMA and ionomycin) and BFA for 6 h. Cells were then fixed, permeabilized and stained with FITC-anti CD3, APC-anti CD4, PE-anti IFN-γ and PE-anti IL-17A mAb, respectively. Intracellular cytokine analysis was performed by flow cytometry. All lymphocytes were first gated based on FSC and SSC. CD3^+^ lymphocytes were then further gated from the lymphocyte cluster according to the fluorescence signal intensities. The results were presented as mean±SEM. (A) The percentage of IFN-γ^+^CD3^+^CD4^+^ cells. (B) The percentage of IFN-γ^+^CD3^+^CD4^-^ cells. (C) Representative flow cytometry plots showing the percentage of IFN-γ^+^CD3^+^ cells in spleen. (D) The percentage of IL-17A^+^CD3^+^CD4^+^ cells. (E) The percentage of IL-17A^+^CD3^+^CD4^-^ cells. (F) Representative flow cytometry plots showing the percentage of IL-17A^+^CD3^+^ cells in spleen. * P<0.05. ns: not significantly different between two groups (p>0.05).

We further characterized cytokine expression profiles of infiltrated T cell population over the course of infection. To enhance the cytokine signals detected by flow cytometry, we conducted the PMA and inomycin treatment. As shown in Fig 5A and 5C, the percentage of IFN-γ^+^CD3^+^CD4^+^ T cells from MAP27-immunized mice (about 7.18±0.96%) was significantly higher than that from MAPctrl-immunized mice (3.85±0.41%, P = 0.021) or blank control mice (3.57±0.73%, P = 0.03). Similar results were also observed for IFN-γ^+^CD3^+^CD4^-^ T cells (Fig 5B and 5C, MAP27 vs MAPctrl, P = 0.021; MAP27 vs blank control, P = 0.012). These results suggest that both CD4^+^ and CD4^-^ T cells were stimulated to produce IFN-γ in MAP27-immunized mice post *S*. *aureus* infection.

Th17 cells have been reported to play an important role in clearance of *S*. *aureus* [14, 38]. Here we also found the percentage of IL-17^+^CD3^+^CD4^+^ T cells from MAP27-immunized mice (about 2.4±0.38%) was higher than that from MAPctrl-immunized mice (about 1.34±0.13%, P = 0.021) and blank control mice (0.31±0.08%, P = 0.012) (Fig 5D and 5F). The percentage of IL-17^+^CD3^+^CD4^-^ T cells is not statistically different among the three groups (Fig 5E). Taken together, not only IFN-γ- but also IL-17-producing T cells of MAP27-immunized mice were stimulated effectively upon *S*. *aureus* infection.

### *In vitro* MAP27 treatment stimulated the splenocytes from MAP27-immunized mice to produce IFN-γ

**Fig 6:**
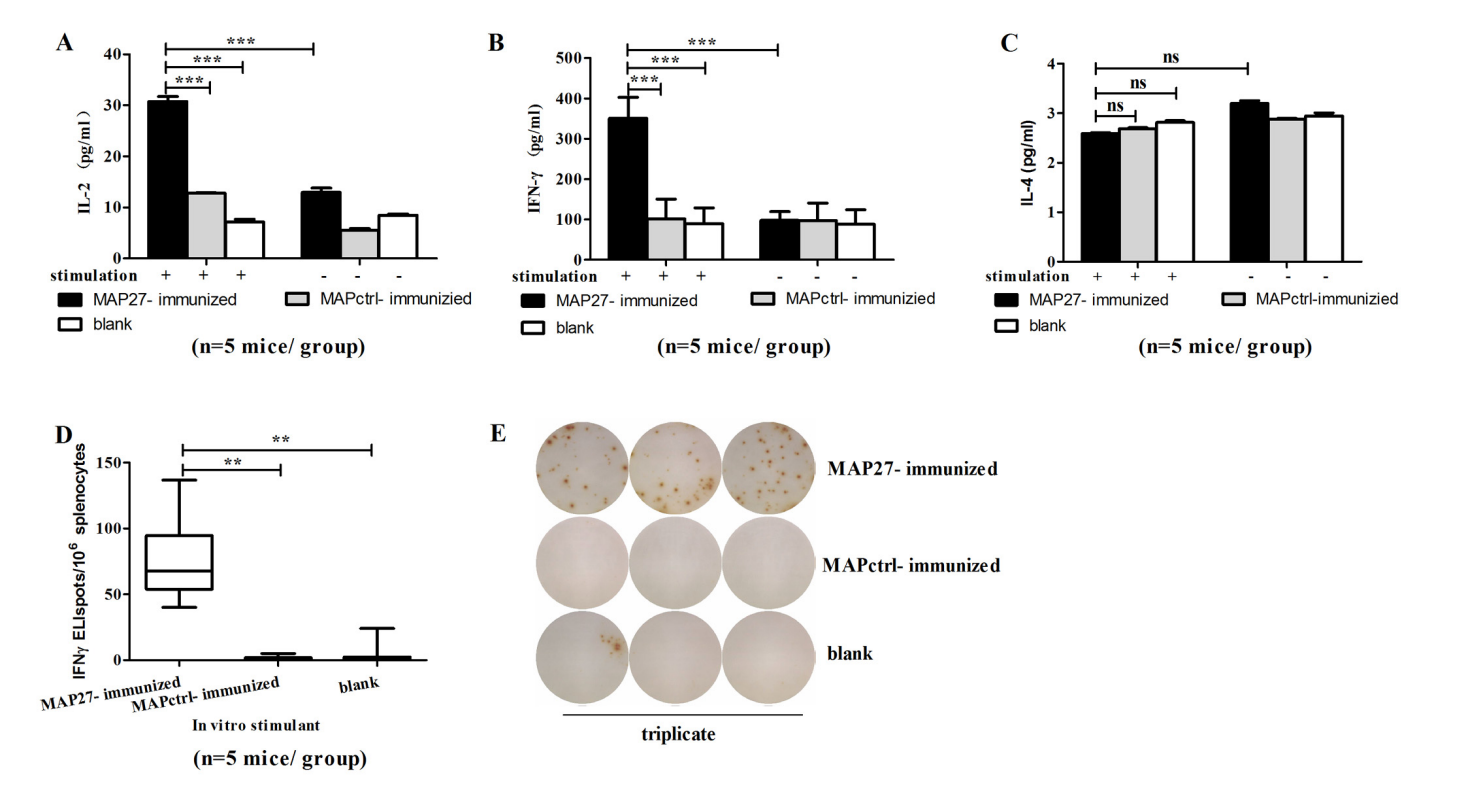
*In vitro* MAP27 treatment promoted the production of IFN-γ in splenocytes from MAP27-immunized mice. Five days after the last boost immunization, splenocytes isolated from the mice (n = 5 mice per group) were cultured at 2×10^5^/well in U-bottom 96-well plates and stimulated with MAP27 (100 μg/ml) for 24 h. The concentrations of IL-2 (A), IFN-γ (B), and IL-4 (C) in supernatant were measured by ELISA. (D) Frequency of IFN-γ-producing cells induced by MAP27. Splenocytes isolated from the mice (n = 5 mice per group) were cultured at 4×10^5^/well in pre-coated plates, and stimulated with MAP27 (10 μg/ml) plus anti-mouse CD28 antibody (1 μg/ml) for 24 h. The number of IFN-γ-producing cells was then measured by ELISPOT and shown in box and whisker plots. (E) Representation of one of the ELISPOT assays. ** P<0.01; *** P<0.001; ns: P>0.05.

To further assess whether immunization with MAP27 was able to induce IFN-γ and IL-17 *in vitro*, we harvested splenocytes after five days of the last boost with heat-inactivated bacteria. Cells were incubated with 100 μg/ml MAP27 for 24 h. The supernatant was then collected and cytokine induction was analyzed by ELISA. The splenocytes from MAP27-immunized mice produced significantly more IL-2 and IFN-γ than cells from control mice upon stimulation by MAP27 (Fig 6A and 6B). In contrast, IL-4, which is mainly produced by Th2 cells, could only be detected very weakly in all three groups (Fig 6C). This can be explained by the fact that high level of IFN-γ in MAP27-immunized mice might suppress Th2 cell differentiation [39]. Unexpectedly, we failed to detect IL-17A/F in supernatant of spleen cells from all the mice (data not shown).

We then used ELISPOT, a more sensitive assay than ELISA, to further determine whether the T cell-mediated response was induced by MAP27. As shown in Fig 6D and 6E, the number of IFN-γ-producing cells from MAP27-vaccinated mice was significantly higher than that from MAPctrl-immunized mice (P = 0.001) or blank control mice (P = 0.001). Again, we could not identify IL-17A-producing cells by ELISPOT assay. In light of this, we speculated that immunization with MAP27 might predominantly induce Th1 cell response.

### Splenocytes from MAP27-immunized mice specifically recognized *S*. *aureus* and produced IFN-γ and IL-17

**Fig 7:**
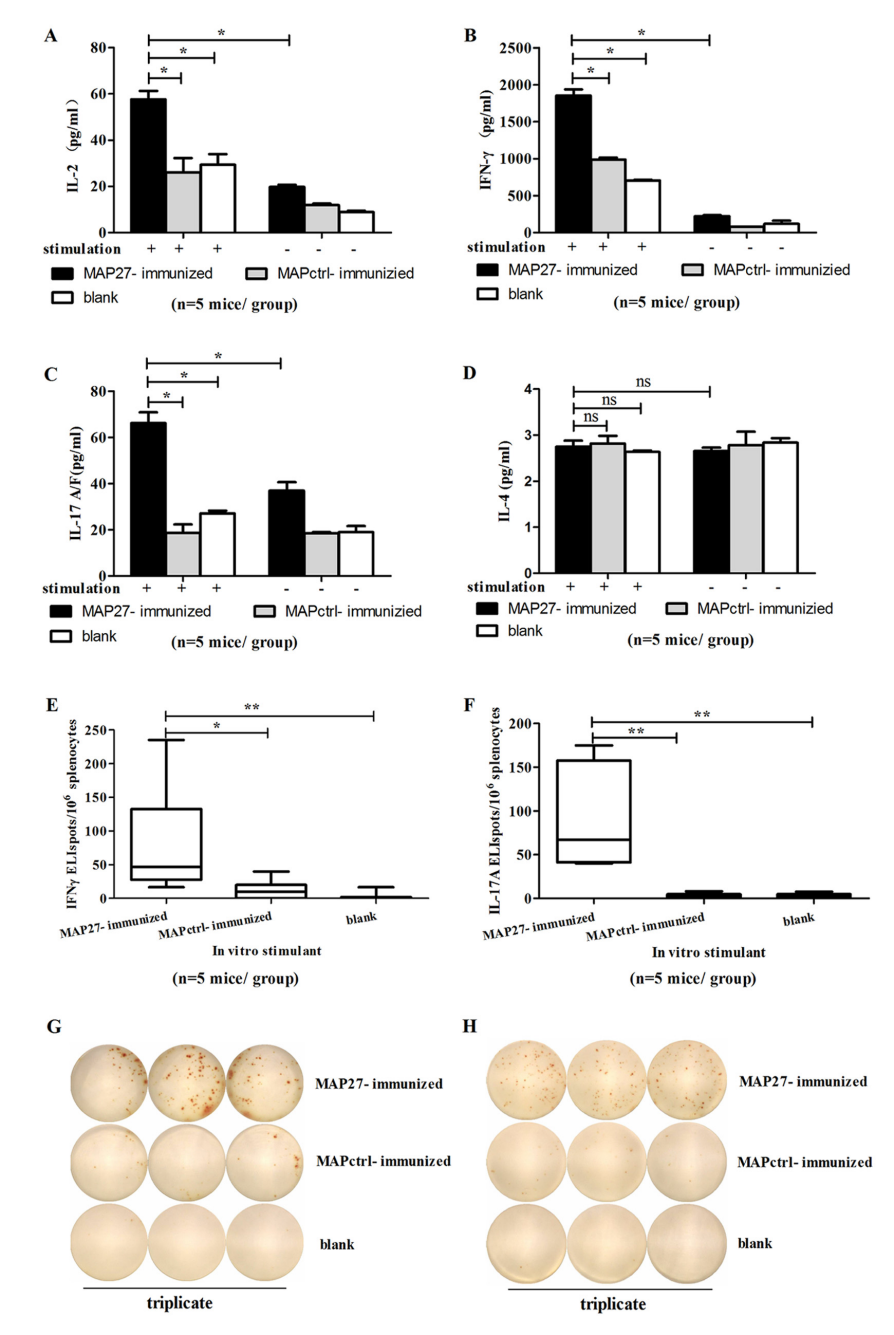
Heat-inactivated *S*. *aureus* promoted Th1 and Th17 cell responses in the spleen of MAP27-immunized mice. Five days after the last booster with heat-inactivated bacteria, the splenocytes isolated from the mice (n = 5 mice per group) were cultured (2×10^5^/well), and stimulated with 2×10^4^ CFU/well heated-inactivated *S*. *aureus* for 72 h. The concentrations of IL-2 (A), IFN-γ (B), IL-17A/F (C) and IL-4 (D) were measured by ELISA. The isolated splenocytes were also cultured at 4×10^5^/well in pre-coated plates and stimulated with heat-inactivated *S*. *aureus* for 24 h. Frequency of IFN-γ-producing (E) and IL-17A-producing cells (F) was determined by ELISPOT. Cell numbers were shown in box and Whisker plots. (G) Representation of one of the ELISPOT assays for IFN-γ-producing cells. (H) Representation of one of the ELISPOT assays for IL-17A-producing cells. * P<0.05; ** P<0.01; ns: P>0.05.

Our next question was whether *S*. *aureus* as a natural antigen could be recognized by splenocytes from MAP27-immunized mice. Splenocytes isolated from three groups of mice were incubated with heat-inactivated *S*. *aureus* for 72 h, and cytokines in the culture supernatant were analyzed by ELISA. Two classical cytokines produced by T cells, IL-2 and IFN-γ, were significantly produced in splenocytes from MAP27-immunized mice, but not MAPctrl-immunized mice or blank control mice (Fig 7A and 7B). Interestingly, IL-17A/F, a cytokine mainly secreted by Th17 cells, is also significantly induced in splenocytes from MAP27-immunized mice (Fig 7C). Similar to the *in vitro* assays with MAP27 stimulation (Fig 6C), IL-4 was not detectable in any group (Fig 7D). We next performed ELISPOT assay to quantitate IFN-γ- or IL-17A-producing cells after *S*. *aureus* stimulation. As shown in Fig 7E–7H, the number of IFN-γ- or IL-17A-producing cells from MAP27-immunized mice was significantly higher than that from the control groups (IFN-γ-producing cells: MAP27 vs MAPctrl, P = 0.0126; MAP27 vs blank control, P = 0.0041; IL-17-producing cells: MAP27 vs MAPctrl, P = 0.0034; MAP27 vs blank control, P = 0.0039). Collectively, these results indicate that T cells, especially IFN-γ- and IL-17-producing T cells in MAP27-immunized mice, recognized effectively the nature antigen *S*. *aureus in vitro*.

## Discussion

To date, the failure to develop an effective vaccine against *S*. *aureus* is partly due to the high complexity of the numerous virulence factors that are expressed at different infection stages. Considering this, Lin and colleagues suggested that immunogens might not be restricted to microbial virulence factors, but any target antigen that effectively induces T cell immune responses could be selected [16]. Here, we used PGN, a conserved cell wall component of *S*. *aureus* as the target, and designed a *de novo* synthetic multiple antigenic peptide (MAP27) for mimicking a PGN epitope. The immunization outcome could be affected by various route of immunization. For example, vaccination of antigens via eyes [40, 41] or oral route [42, 43] has been shown to induce specific tolerance in BALB/c mice. In this study, we found that subcutaneous immunization, with peptides emulsified with Freund’s adjuvant, effectively induced immune response and protected the mice against *S*. *aureus* systemic infection. It would be very interesting to test whether other routes of immunization, such as via mucosal immunization, could further enhance the protective effect.

As shown in Fig 2B, immunization with MAP27 alone could only weakly induce IgG to bind to PGN. This can be explained by the fact that the peptide only mimics a single epitope of PGN, and possibly the peptide mimicking is not completely identical to the natural antigen. Moreover, as an artificial antigen, MAP27 probably has weaker immunogenicity compared to most of the protein immunogens. Since previous findings have showed that boosting with natural antigen after peptide immunization could induce a robust recall response [33, 34], we modified our immunization strategy by boosting the mice with heat-inactivated *S*. *aureus* after MAP immunization. Our results demonstrated that boosting with natural antigen improved the binding of the sera to the bacteria (Fig 2C). In contrast, the same boosting step had no effect on the control groups (Figs 2C and 3A). This result is consistent with the previous finding that vaccination with heat-killed *S*. *aureus* before infection does not affect bacterium outcome [44].

Although the titer of sera against PGN or *S*. *aureus* is low, MAP27-immunization could still protect the mice from *S*.*aureus* systemic infection (Fig 3). Previous studies have demonstrated that IL-17 and Th17 are critical for protection from *S*.*aureus* infection [9, 11, 14]. Moreover, IFN-γ is universally considered to improve the survival of staphylococcemia mice by activating marcrophages and neutrophils to kill bacteria [45]. M1-polarized macrophages stimulated with IFN-γ could also inhibit the formation of *S*. *aureus* biofilm [46]. Based on these facts, we hypothesized that the protection is possibly through the activation of T cell-mediated responses. Several evidences to support this hypothesis were presented in this study: 1) Levels of IFN-γ and IL-17A/F were significantly increased in the early stage of bloodstream infection (Fig 4); 2) Both IFN-γ^+^CD3^+^CD4^+^ Th1 cells and IL-17^+^CD3^+^CD4^+^ Th17 cells significantly increased in MAP27-immunized mice after infection (Fig 5); 3) Our *in vitro* assay showed that splenocytes from MAP27-immunized mice specifically recognized *S*. *aureus* and produced IFN-γ and IL-17 (Fig 7). Interestingly, we found that IFN-γ^+^CD3^+^CD4^-^ T cells also increased in MAP27-immunized mice post infection (Fig 5B and 5C). We speculated that MAP27 immunization might induce different subsets of T cells to secrete IFN-γ post infection. For example, CD8^+^ T and γδ T cells have been reported to secrete IFN-γ and protect mice from *S*. *aureus* systemic infection [17, 47, 48].

Different from heat-inactivated *S*. *aureus* stimulation (Fig 7), MAP27 failed to induce IL-17A/F in splenocytes (data not shown), even though it induced high level of IFN-γ *in vitro* (Fig 6B). Again, this result can be explained by the fact of the inhibitory role of IFN-γ in Th17 differentiation [49]. We speculated that the artificial MAP antigen and the natural antigen (*S*. *aureus*) might trigger different responses. However, the exact mechanism is still unclear and needs to be elucidated. A previous study suggested that MAP can be internalized by antigen presenting cells (APCs) and presented as an epitope on cells [50]. It would be very interesting to investigate how APCs recognize this peptide to elicit T cell responses.

In summary, our results indicate that immunization with PGN mimicking peptide, MAP27, could protect mice against *S*. *aureus* systemic infection. This protection might be related with the stimulation of IFN-γ- and IL-17-producing T cells that specifically recognized *S*.*aureus*.

## Supporting Information

S1 Fig MAP27 immunization decreases the bacterial burden in organs. Mice were immunized with MAP27 or MAPctrl for five times at a two-week interval without bacterial boost (A, C), or with bacterial boost (B, D). All the mice were infected with *S*. *aureus* via tail vein post five days of the last immunization. The bacterial numbers in organs were measured after three days of infection. Bacterial burden in kidney (A, B) and in lung (C, D) were measured. (* P<0.05; ** P<0.01) (n = 4-11/group).
(TIF)
