# Supplementary figures and images for: A Multiple Antigenic Peptide Mimicking Peptidoglycan Induced T Cell Responses to Protect Mice from Systemic Infection with Staphylococcus aureus
Source: PLoS One. 2015 Aug 28;10(8):e0136888. doi: 10.1371/journal.pone.0136888 (PMC4552945; doi:10.1371/journal.pone.0136888)

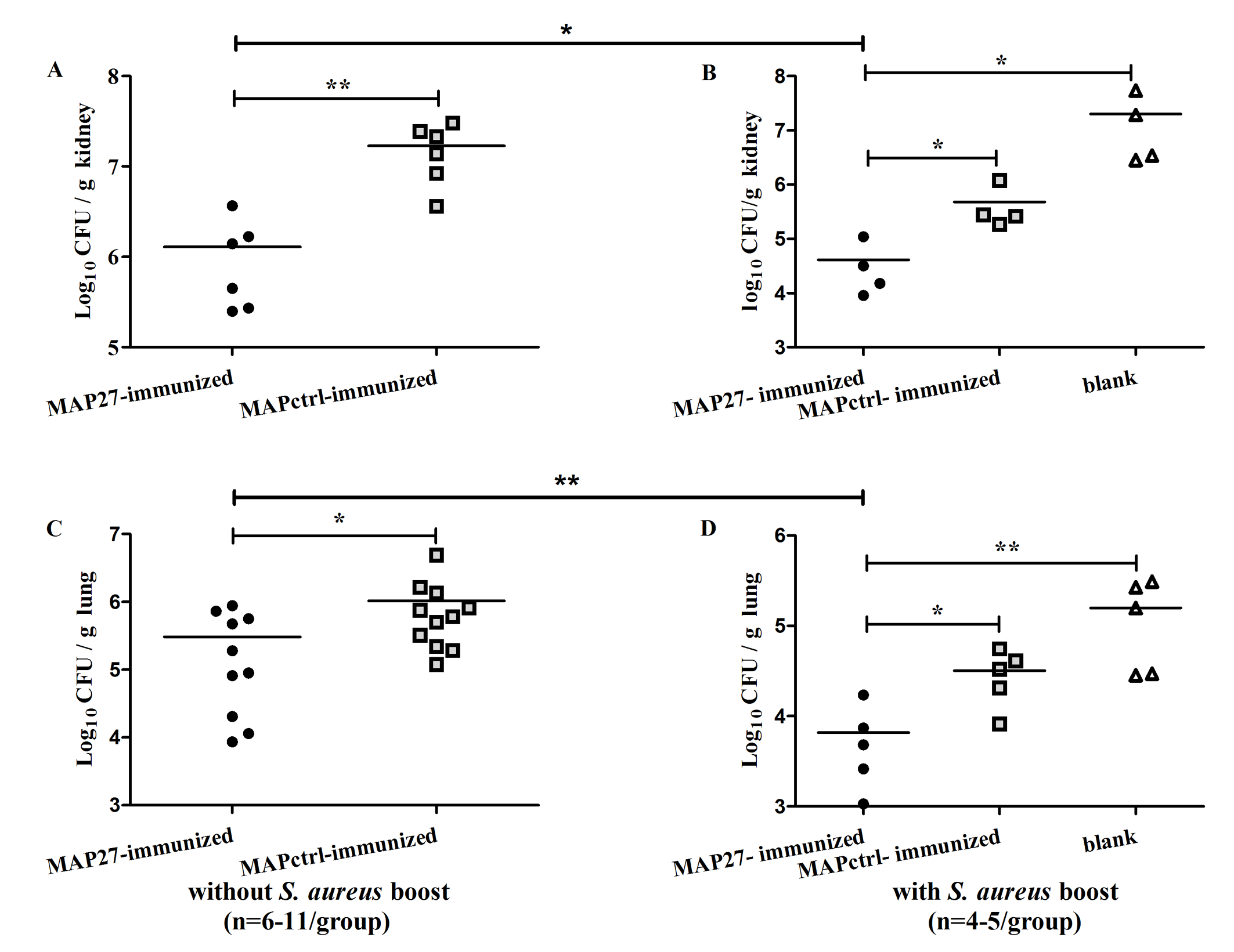

Supplement: S1 Fig — Mice were immunized with MAP27 or MAPctrl for five times at a two-week interval without bacterial boost (A, C), or with bacterial boost (B, D). All the mice were infected with S. aureus via tail vein post five days of the last immunization. The bacterial numbers in organs were measured after three days of infection. Bacterial burden in kidney (A, B) and in lung (C, D) were measured. (* P<0.05; ** P<0.01) (n = 4-11/group). (TIF) [file pone.0136888.s001.tif]
